# An Account of Some Experiments to Prove the Advantage of Employing the Alcoholic Solution of Guiaic, as a Test of the Goodness of the Dried Bulb of Colchicum Autumnale; with Some Remarks on the Best Period for Taking up the Bulb, and the Method of Drying It so as to Preserve Its Medicinal Properties Unaltered

**Published:** 1820-10

**Authors:** Anthony Todd Thomson


*An Account of some Experiments to prove the Advantage of employing the Alcoholic Solution of Guiaic, as a Test of the Goodness of the dried Bulb of Colchicum Autumnale; with some Remarks on the best Period for taking up the Bulb, and the Method of drying it so as to preserve its Medicinal Properties unaltered.* By ANTHONY TODD THOMSON, F.L.S. &c.

THERE is a fashion in medicine, which has occasionally expelled from practice some of the best articles of the materia medica, and held them in disrepute, until accident has again restored them to favour. Such has been the fate of the meadow saffron, *colchicum autumnale*, a very ancient, and one of the most manageable and efficacious remedies, in many painful and inflammatory affections, when it is good of its kind and properly prescribed; and such is likely again to be its lot, if some means be not devised to enable practitioners readily to discover when it is in a condition fit to be employed; for the state in which it is now kept in the majority of the shops of the London druggists, and even at Apothecaries' Hall, can only produce disappointment to the physician, who may prescribe it in cases which require the full extent of its powers. That the reliance of many practitioners upon it has already been weakened by these circumstances, I have had several opportunities of knowing; and the wish to restore that faith in its virtues which the remedy truly deserves, has suggested the propriety of laying the observations and experiments about to be detailed before the profession.

Whilst lately repeating the experiments of M. Taddei on gluten, I was astonished to find how readily that principle is destroyed, in many plants which contain it, by the action of heat; and, reflecting on the changes which necessarily take place in all the components of a vegetable body when one of them is destroyed, I was naturally led to infer, that those specimens of any medicinal plant, in which the components can be preserved in a state the nearest to that which they hold before it is dried, must be the most efficacious and certain in their remedial properties. I had ascertained the correctness of M. Planche's assertion, that gluten is one of the constituents of the recent bulb of meadow saffron, and discovered that its presence can easily be detected in the dried bulb; and, as I had found that those specimens of it in which this principle can be demonstrated are the most powerful in their effects, I inferred that these only should be regarded as properly dried, and fit to be medicinally employed in which it is found, provided they bear marks of having been dug up at a proper season; and, therefore, that the agent by which gluten is detected in the dried bulb of colchicum, must be regarded as a proper test of its goodness. But, before making experiments to prove this opinion, it was necessary to fix the exact period at which the bulb should be dug up; and that this is towards the end of July, some observations I had been making on the physiology of bulbs had already enabled me to determine. The bulb of the last year is then completely decayed, and its progeny, those of the present year, are at their full size and in their most perfect condition. When dug up, each bulb is found covered with a smooth, shining, thick, mahogany-brown coat; it is plump, solid, and in shape somewhat resembling a roundish pear; the withered remains of the foliage answering to the stem, with a sharp ridge partially surrounding the base, and terminating in a short appendix. On removing this coat, and a spongy one which is interior to it, a small white oval body, about a line in breadth, one-third of an inch in length, and slightly elevated, is seen seated in a depression near the point of the appendix, on one side; and another of the same description, a little higher up, in another slight depression, on the opposite side of the bulb. These are the rudiments of the expected flowers and leaves; and, when the sheath by which they are covered has attained an inch in length, the bulb has already begun to lose its medicinal qualities. If it be taken up in July, and sliced transversely, each slice has a nearly circular figure, slightly approximating to the oval, but perfectly free from any indentation on either side; and this form is retained in the dried slice: whereas, when the flowers appear, every slice is panduriform, or an oblong oval, with a deep semicircular notch or depression on each side; and every approach to this figure implies that the bulb has been dug up too late in the season. These indentations arise from the sheaths of the flowers, as they enlarge, pressing upon the sides of the bulb, which now readily yield, owing to the absorption of its contents for the support of the growing flowers first taking place in the part immediately under that on which the sheath presses. At that part the exhausting process is immediately recognized, by the spongy appearance which is seen surrounding each indentation, when the bulb is transversely divided.

Having made these preliminary enquiries, I obtained nine specimens of dried colchicum for my experiments.

*Experiment* 1.—Ten grains of the bulb of colchicum, which was dug up on the 25th of July, and dried in transverse slices less than one-quarter of an inch in thickness, *without heat*, were rubbed in a mortar with sixteen minims of distilled vinegar, and immediately afterwards with sixteen minims of an alcoholic solution of guiaic: a most beautiful cærulean blue was almost directly evolved, and remained permanent until the following day, when the mortar was cleaned.

The specimen subjected to this experiment was taken up by myself; and, in drying it, the slices were simply spread out, each slice separate from another, on a sheet of clean white paper, placed in an airy situation excluded from the sun. The dried slices were very little shrunk, of a cream-white, opaque, and somewhat granular on both surfaces; perfectly friable, devoid of colour, and bitter to the taste, without the least trace of sweetness. Three grains of the powder produced some degree of nausea, purged moderately, and diminished both the quickness and strength of the pulse. It was therefore marked No. 1, and regarded as the criterion for determining the quality of the other specimens.

I ought to mention that, in all the experiments with the dried bulb, the powder was first rubbed with distilled vinegar; because I had ascertained that, in dry amylaceous powders containing gluten, this principle is in a state which prevents it from being readily acted on by the guiaic, unless it be previously dissolved; and, as acetic acid is the best solvent of gluten, distilled vinegar was employed for that purpose. Distilled vinegar, when added alone to the alcoholic solution of guiaic, merely precipitates the guiaic unaltered; consequently, the change of colour cannot in any degree be ascribed to that agent; and the same colour is evolved if the powder of colchicum be well rubbed with the solution alone, although it is by no means so quickly produced.

*Experiment* 2.—Ten grains of dried colchicum, obtained at Apothecaries' Hall, were rubbed with sixteen minims of distilled vinegar, and immediately afterwards with sixteen minims of the alcoholic solution of guiaic; but no blue colour was evolved. The friction was continued, and a few drops more of the vinegar and the alcoholic solution were added; but, at the end of twenty minutes, no blue colour was perceptible.

In this specimen (No. 2), the bulbs, with the exception of four thin slices in one ounce, (the quantity purchased at Apothecaries' Hall,) were entire, shrivelled, and deeply marked with the indentations which indicate that the bulb has been in flower, or just about to flower, at the time it is taken up; while several of the bases of the flowering sheaths, in an apparently charred state, were also present. The bulbs, when sliced, instead of being white, were of a light brown or rather fawn colour, tough, with a peculiar odour, not unlike that of charred malt; and both bitter and sweet to the taste. Indeed, in some of them the sweetness predominated, and the odour was much increased in the powder. Eight and ten grains taken into the stomach did not nauseate, scarcely purged in some cases, and in others not at all, and did not produce any very sensible effect on the pulse.

Although I have no means of knowing the fact, yet I am inclined to imagine that the people employed to collect the bulbs for the Apothecaries' Company, being ignorant where to find them until the flowers show themselves above ground, wait until that period for taking them up; and that they are further deteriorated by being dried in a stove, at a temperature sufficient to destroy the gluten, and consequently to change the other components of the bulb so much as to diminish in a very considerable degree its medicinal properties. The method of preventing the drying from injuring the bulbs, has been already mentioned; and I would suggest their cultivation, which I know from experience does not diminish their medicinal properties, as the best mode of obviating the disadvantage which results from not being able to collect them in their wild state until the flowers appear.

*Experiment* 3.—Ten grains of a specimen of dried colchicum, procured at the shop of Messrs. Watts and Co. Strand,* being treated exactly in the same manner as No. 1, almost instantaneously evolved a deep-blue colour; but, however, in three minutes it changed to a glaucous green, and after some time became considerably darker, or sea-green.

In this specimen (No. 3), the bulbs, although in a better state

* It is necessary to mention that, with the exception of Mr. Battley, I am not acquainted with any of the druggists from whom the specimens submitted to experiment were purchased.

than those of No. 2, yet bore evident marks of having been taken up when the flower was advanced. They were rather small, split longitudinally in halves, and had been apparently dried without heat; in their substance friable, inodorous, and much more bitter, with scarcely any of the sweetness perceptible in those of No. 2. The powder was of a very light fawn hue; but, although the bulb was in a condition to produce its effects in an increased dose, it was evident that it had been collected in too advanced a state.

*Experiment* 4.—Ten grains of a specimen procured from the shop of a highly respectable druggist at the west end of the town,* and treated in the same manner as No. 1, produced no change of colour, after twenty minutes' rubbing.

This specimen (No. 4), was in a worse state than that of No. 2, some of the bulbs being quite black.

*Experiment* 5.—Ten grains of a specimen procured from another highly respectable druggist in the City, and treated as No. 1, evolved, after three minutes' rubbing, a glaucous green, which after some time became paler, and passed almost into a grey.

In this specimen (No. 5), the bulbs were also split longitudinally. Some of the pieces were firm, and internally white and good; but others were quite spongy, and displayed, when broken, the vessels of the bulb in a ligneous state, which occurs only after the flower has decayed, and the leaves are appearing in early spring. They had little odour, and the taste was bitter devoid of any sweetness, which led me to think no artificial heat had been employed in drying them; but the pieces appeared as if they had been kept for several years. The powder was of a light-fawn colour.

*Experiment* 6.—Ten grains of a specimen obtained from another respectable druggist in the City, and treated as No. 1, evolved immediately a very deep bluish green; which however very soon faded.

In this specimen (No. 6), the bulbs were also split longitudinally, and many of the pieces were in the same state as the best of those in No. 5; but the others were spongy. One of them, indeed, was not a colchicum bulb, but that of *crocus autumnalis.* The powder of this specimen was a very pale fawn colour, nearly white; it had no odour, and scarcely any sweetness; but neither was the bitter very perceptible.

*Experiment* 7.—Ten grains of a specimen procured at Butler's, in Covent Garden, and treated as No. 1, produced

* As it is not my wish to injure the reputation of any one, I forbear from mentioning the names of those druggists from whom the bad specimens were procured.

almost immediately a greenish-blue colour; but certainly with less of the green than displayed by Nos. 4, 5, and 6. It became paler after a short time.

In this specimen (No. 7), the bulbs were sliced transversely, but the slices were too thick, and consequently tough instead of being friable, and a few of them were spongy. The taste was bitter without any sweetness; and the specimen bore the appearance of having been dried without artificial heat. Indeed, with the exception of the marks of having been taken up at too advanced a season, it was a good sample of the drug.

*Experiment* 8.—Ten grains of a specimen from the shop of Messrs. Herring and Co. treated as No. 1, afforded almost immediately a light blue, without any tint of green; and the colour did not much fade when left for some hours.

In this specimen (No. 8), the bulbs were split longitudinally, but they were also firm, friable, internally white, inodorous, and impressed the proper bitter taste of the bulb without any sweetness. They were, however, too deeply notched; but, in other respects, the specimen was an excellent sample of the drug, and had been apparently dried without artificial heat, or at a very low temperature.

*Experiment* 9.—Ten grains of a specimen procured from Mr. Battley, of Fore-street, Cripplegate, treated in the same manner as No. 1, after two minutes' hard rubbing, afforded a pale blue, without the smallest tint of green, and became darker when left in the mortar for some hours.

This specimen was in transverse slices, about the thickness of the tenth of an inch, of a cream-white colour, opaque, and friable, with the granular surface mentioned in No. 1, but notched; some of the slices being of a kidney-shape, and others panduriform. It was free from odour, the bitter not strong; and, although it was devoid also of sweetness, yet it had something of the flavour of the sweet almond. As Mr. Battley has published his mode of drying the bulb, I am of opinion that the temperature of 170 to 180, to which he exposes it, is even too high; and that, although the gluten be not entirely destroyed, yet it is partially so; which accounts for the blue colour being longer in appearing, and for the almond taste it impresses when chewed: and, if my inference be correct, that all the components should be in a state nearly the same as before the bulb is dried, to secure the full medicinal qualities of the drug, it is evident that, as even so low a temperature as 170 is in some degree destructive of the balance of the components which is maintained in the recent colchicum, it should not be employed. In every other respect this specimen was excellent, and approached nearer to the quality of No. 1, than any of the others.

From these experiments, and the prefatory observations, we are fully authorized in concluding,

1. That the diversity of opinions, held by practitioners at the present time, regarding the medicinal powers of the bulb of colchicum autumnale, proceed from the different conditions in which it is found in the shops, and in which it is consequently administered.

2. That the month of July is the best period for taking up the plant, as the bulb has then attained its full growth and perfection; whilst the vegetation of the gems or lateral progeny, for the support of which the bulb is intended, has scarcely commenced.

3. That the bulb, when taken up, should as soon as possible be cut, as Mr. Battley directs, "into transverse slices, equal in thickness to half-a-crown;"* and these, being spread out upon clean white paper, should be dried without artificial heat, in an airy situation screened from the sunshine.

4. That the slices, when dried, should be nearly oval, but not notched or panduriform; friable, of a white or cream colour, somewhat granular on both surfaces; inodorous; bitter to the taste, and altogether free from sweetness; and should afford a fine cærulean blue colour, when rubbed with a few drops of vinegar and the alcoholic solution of guiaic.

5. That practitioners who compound their own prescriptions, should purchase the drug in the sliced state, and test it in the manner above described before employing it.

6. That a physician, in prescribing the remedy, should endeavour to ascertain in what condition it is kept in the shop of the druggist who is to compound his prescription; and that the Royal College of Physicians should notify to the Apothecaries' Company, that, owing to the condition in which the dried colchicum is now sold in their Hall, it is not capable of effecting the object for which it is prescribed.

*Sloane-street; September* 1820.

* Vide *London Medical Repository*, vol. xiv. p. 29.

